# Supplementary figures and images for: Members of the DIP and Dpr adhesion protein families use cis inhibition to shape neural development in Drosophila
Source: PLoS Biol. 2025 Mar 3;23(3):e3003030. doi: 10.1371/journal.pbio.3003030 (PMC12135937; doi:10.1371/journal.pbio.3003030)

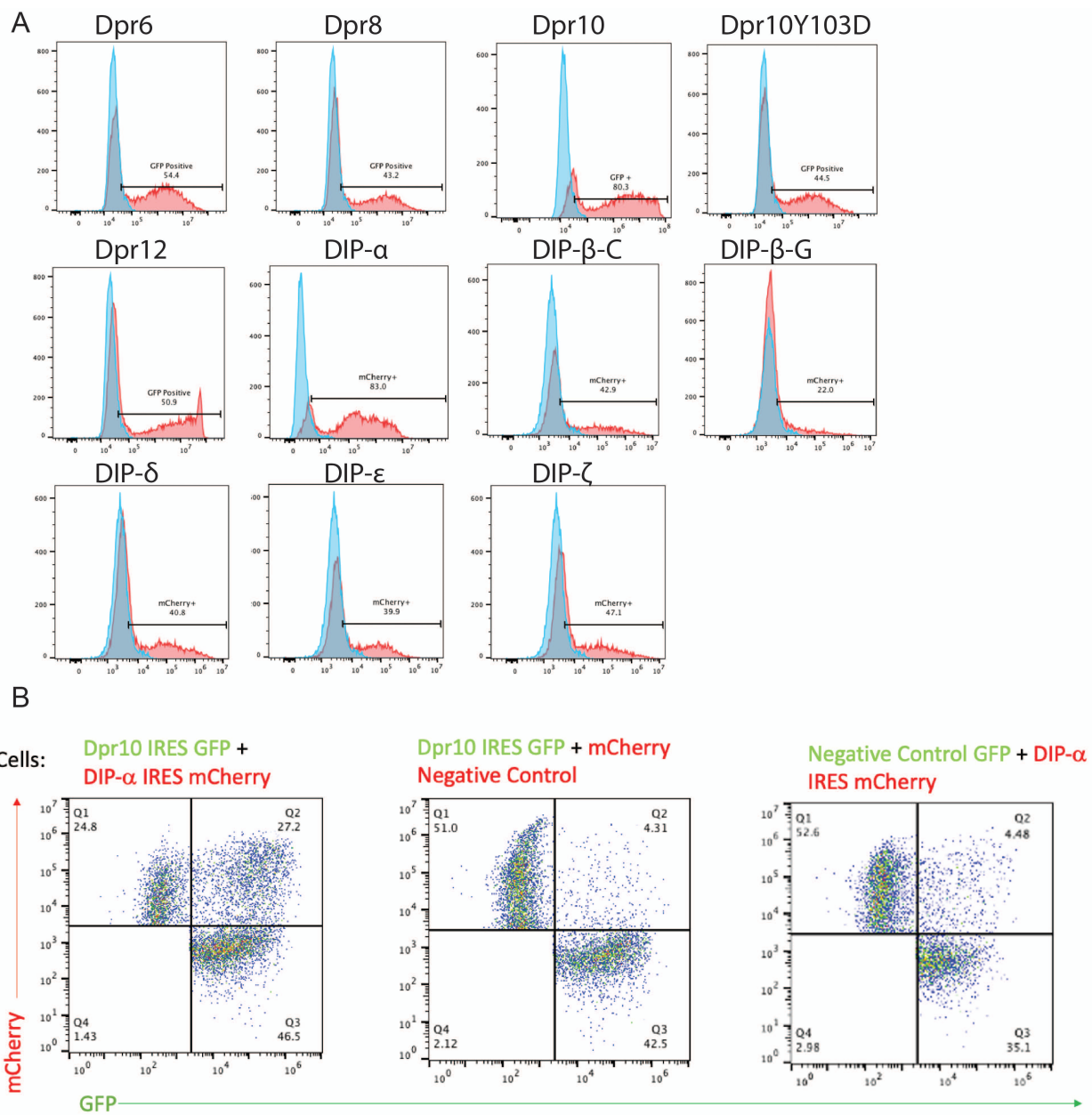

**Figure S1:**

Supplement: S1 Fig — (A) Percent positive of mCherry (DIPs) and GFP (Dprs) expressed as full-length proteins with an IRES tag in mammalian cells. (B) Representative flow plots showing typical binding and signal to noise for Dpr10::DIP-α cell-cell adhesion. (PDF) [file pbio.3003030.s001.pdf]

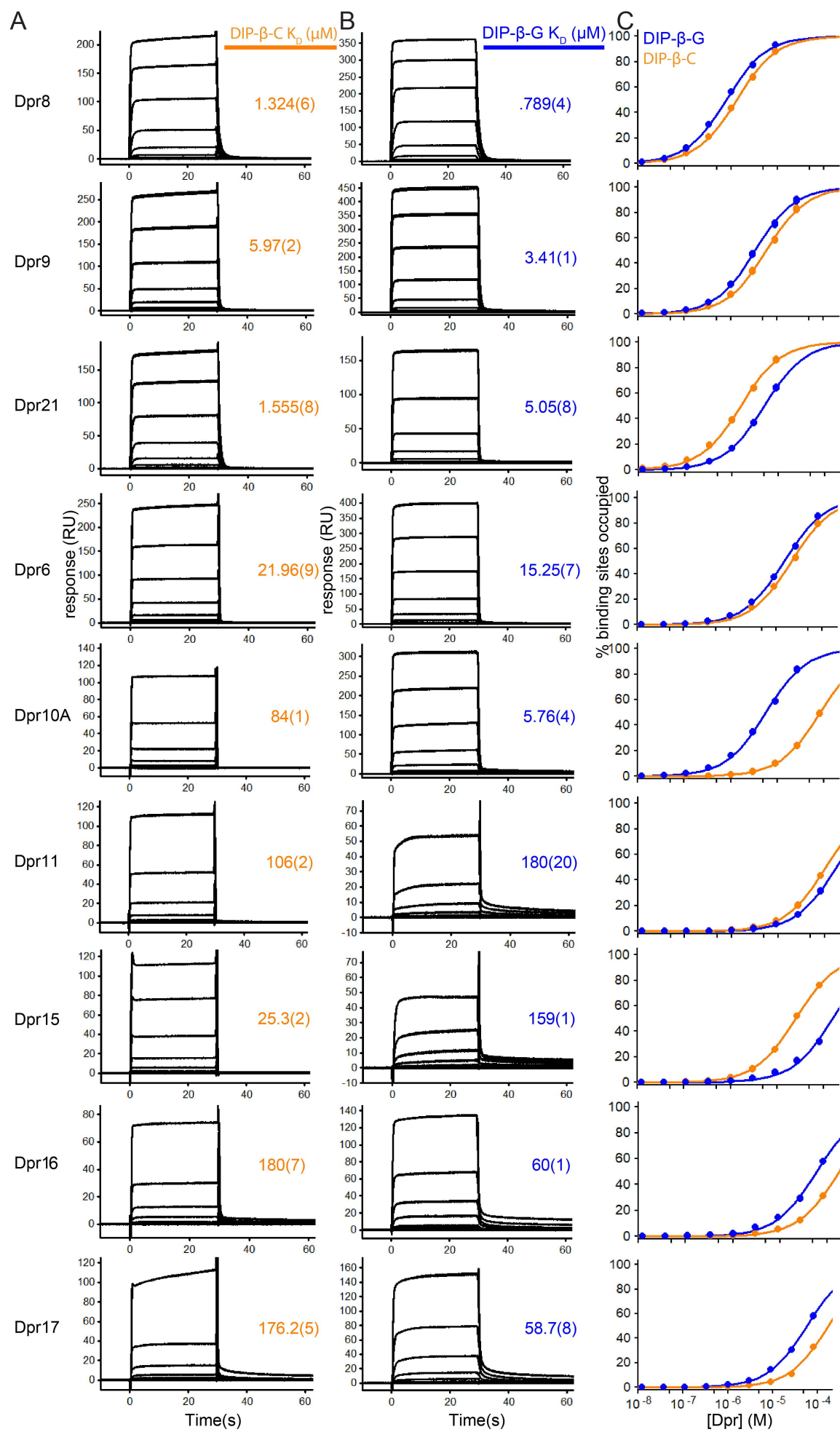

**Figure S2:**

Supplement: S2 Fig — (A) Sensorgrams of Dpr analytes binding over DIP-β-G immobilized surface and (B) Sensorgrams of Dpr analytes binding over DIP-β-G immobilized surface. (C) The fit of the binding data to 1:1 binding isotherms to calculate KD. Numbers in parentheses indicate error. The raw data underlying these figures can be found in S8 Data. (PDF) [file pbio.3003030.s002.pdf]

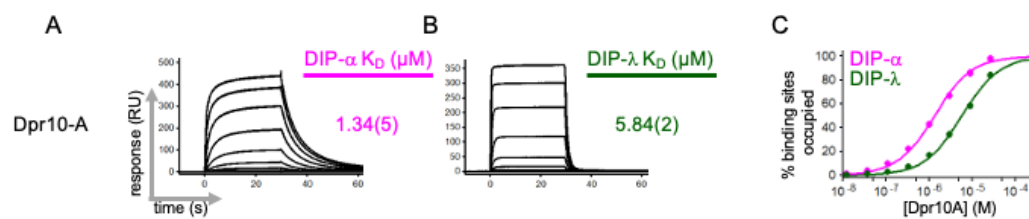

**Figure S3:**

Supplement: S3 Fig — (A) Sensorgrams of Dpr10-A analytes binding over DIP-ɑ immobilized surface. (B) Sensorgrams of Dpr10-A analytes binding over DIP-λ immobilized surface. (C) The fit of the binding data to 1:1 binding isotherms to calculate KD. Numbers in parentheses indicate error. The raw data underlying these figures can be found in S8 Data. (PDF) [file pbio.3003030.s003.pdf]

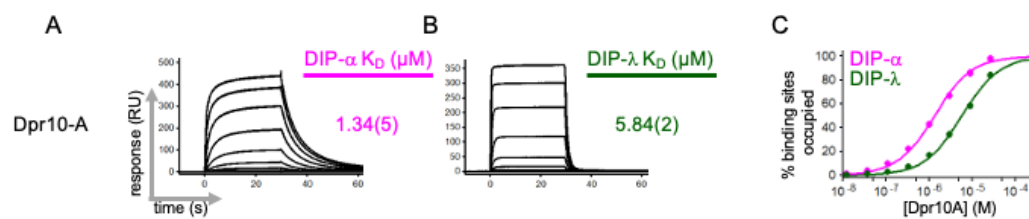

Figure S3:

Supplement: S4 Fig — (A) Flow plots showing the gating strategy for analyzing co-transfected cells and representative plot of efficiency of co-transfecting cognate DIPs (mCherry- X axis)) and (Dprs- GFP- Y-axis). (B) Representative flow plots showing binding differences of 300 nM Dpr10-Fc to DIP-ɑ expressing cells or (C) DIP-ɑ/Dpr10 co-expressing cells. (D) Representative flow plots showing binding differences of 300 nM Dpr12-Fc to DIP-δ expressing cells or (E) DIP-δ/Dpr12 co-expressing cells. (PDF) [file pbio.3003030.s004.pdf]

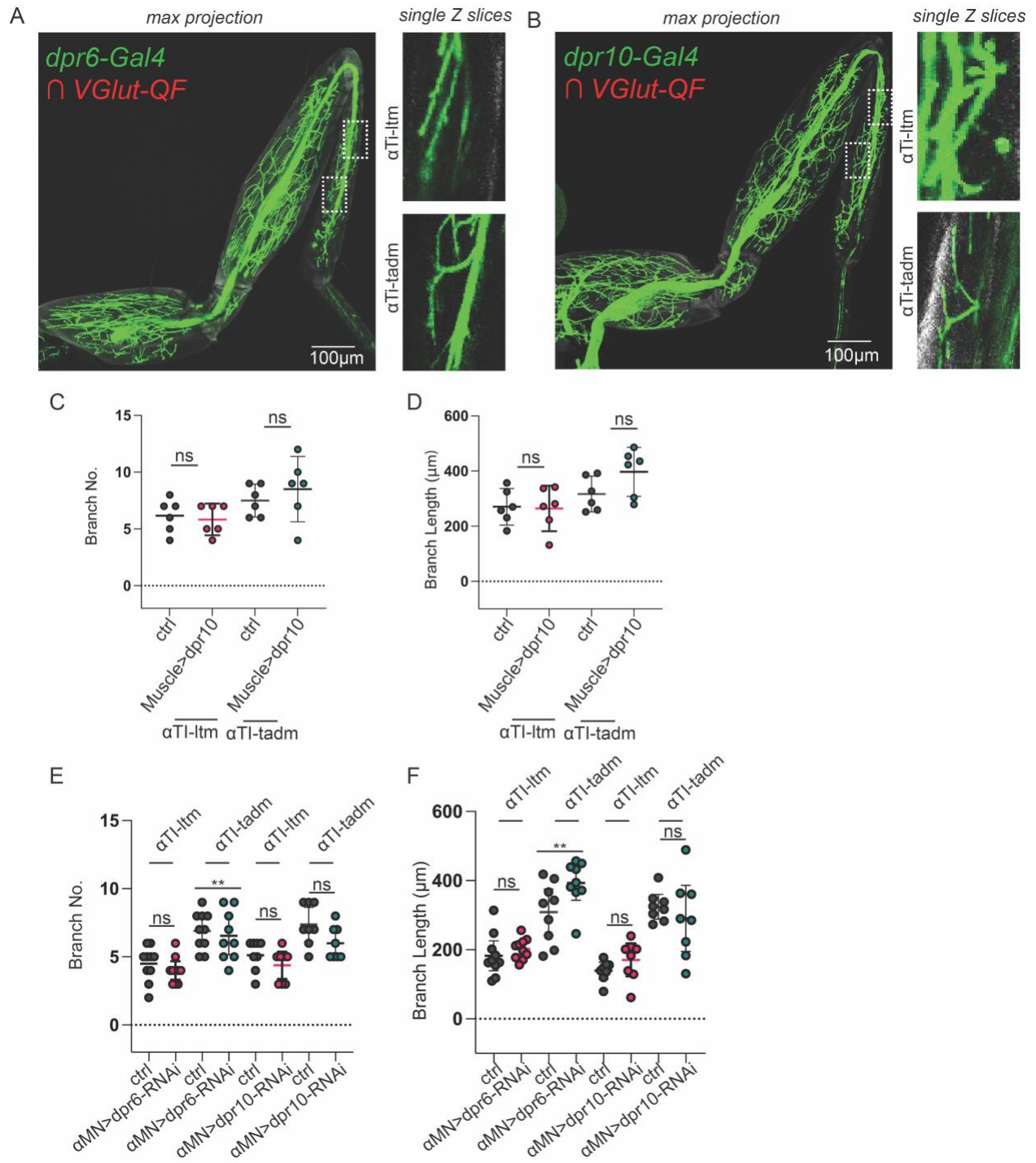

**Figure S7:**

Supplement: S7 Fig — (A) Quantification of the number of αTi-ltm and αTi-tadm terminal branches. Ctrl, DIP-α-T2A-QF>>10XUAS6XGFP. αTi-ltm n = 6. αTi-tadm n = 6 Muscle>dpr10, DIP-α-T2A-QF>>10XUAS6XGFP, Mef2-Gal4 > UAS-dpr10. αTi-ltm n = 6. αTi-tadm n = 6. (B) Quantification of the sum of αTi-ltm and αTi-tadm terminal branch length. Ctrl, DIP-α-T2A-QF>>10XUAS6XGFP. αTi-ltm n = 6. αTi-tadm n = 6 Muscle>dpr10, DIP-α-T2A-QF>>10XUAS6XGFP, Mef2-Gal4 > UAS-dpr10. αTi-ltm n = 6. αTi-tadm n = 6. (C) Quantification of the number of αTi-ltm and αTi-tadm terminal branches. Ctrl, DIP-α-T2A-Gal4>>20XUAS6XGFP. αTi-ltm n = 10. αTi-tadm n = 10 αMN>dpr6-RNAi, DIP-α-T2A-Gal4>>20XUAS6XGFP,UAS-dpr6-RNAi. αTi-ltm n = 10. αTi-tadm n = 10 Ctrl, DIP-α-T2A-Gal4>>20XUAS6XGFP. αTi-ltm n = 8. αTi-tadm n = 8 αMN>dpr10-RNAi, DIP-α-T2A-Gal4>>20XUAS6XGFP,UAS-dpr10-RNAi. αTi-ltm n = 8. αTi-tadm n = 8. (D) Quantification of the sum of αTi-ltm and αTi-tadm terminal branch length. Ctrl, DIP-α-T2A-Gal4>>20XUAS6XGFP. αTi-ltm n = 10. αTi-tadm n = 10 αMN>dpr6-RNAi, DIP-α-T2A-Gal4>>20XUAS6XGFP,UAS-dpr6-RNAi. αTi-ltm n = 10. αTi-tadm n = 10 Ctrl, DIP-α-T2A-Gal4>>20XUAS6XGFP. αTi-ltm n = 8. αTi-tadm n = 8 αMN>dpr10-RNAi, DIP-α-T2A-Gal4>>20XUAS6XGFP,UAS-dpr10-RNAi. αTi-ltm n = 8. αTi-tadm n = 8. For all graphs, statistical significance was determined using an unpaired nonparametric two-tailed Mann–Whitney test. Error bars represent mean with 95% confidence intervals. ns = no statistical difference. *p < 0.05, **p < 0.01, ***p < 0.001, ****p < 0.0001. The raw data underlying these figures can be found in S9 Data. (PDF) [file pbio.3003030.s007.pdf]

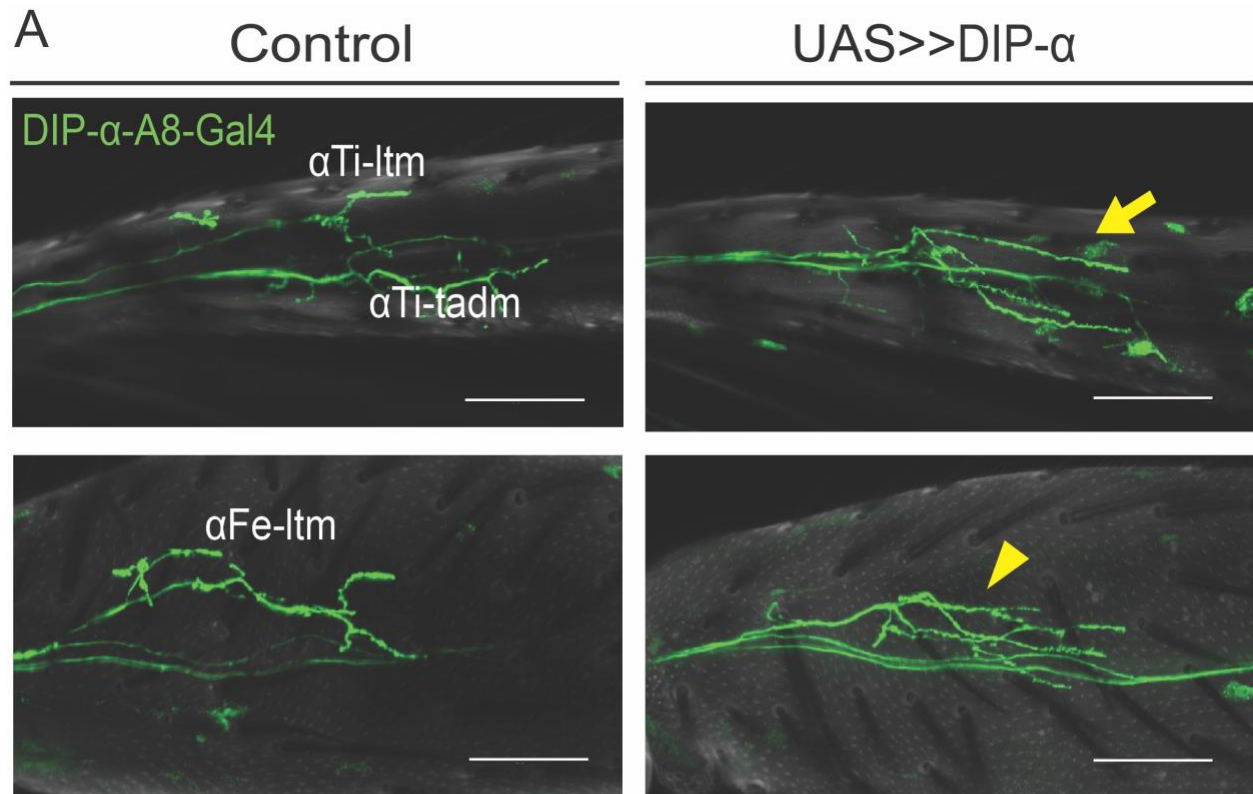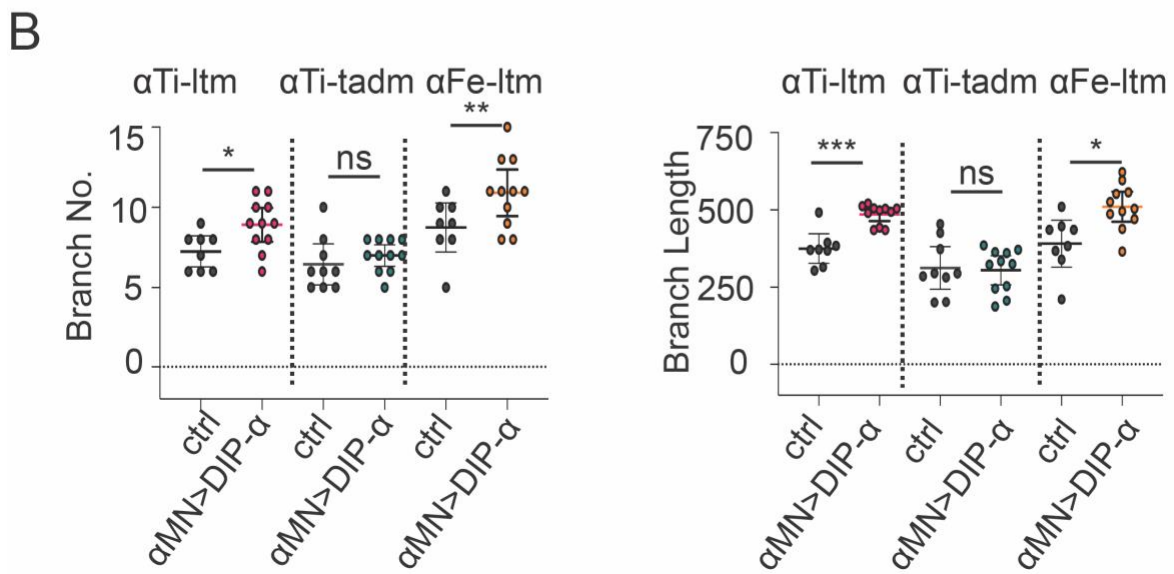

**Figure S8**

Supplement: S8 Fig — A) Representative images of DIP-α-A8-Gal4>>20XUAS6XGFP controls, and DIP-α-A8-Gal4>>20XUAS6XGFP, UAS- DIP-α. Yellow arrow indicates longer αTi-ltm axons. Yellow arrowhead indicates longer αFe-ltm axons. Scale bar = 50µm. (B) Quantification of the number of αTi-ltm, αTi-tadm and αFe-ltm terminal branches.Ctrl, DIP-α-A8-Gal4 20XUAS6XGFP. αTi-ltm n = 8. αTi-tadm n = 9 αFe-ltm n = 8. DIP-α-A8-Gal4>>20XUAS6XGFP, UAS-DIP-α. αTi-ltm n = 11. αTi-tadm n = 11 αFe-ltm n = 11. Quantification of the sum of αTi-ltm, αTi-tadm, and αFe-ltm, branch length. Ctrl, DIP-α-A8-Gal4 20XUAS6XGFP. αTi-ltm n = 8. αTi-tadm n = 9 αFe-ltm n = 8. DIP-α-A8-Gal4>>20XUAS6XGFP, UAS-DIP-α. αTi-ltm n = 11. αTi-tadm n = 11 αFe-ltm n = 11. For all graphs, statistical significance was determined using an unpaired nonparametric two-tailed Mann–Whitney test. Error bars represent mean with 95% confidence intervals. ns = no statistical difference. *p < 0.05, **p < 0.01, ***p < 0.001. Ctrl-Control, MN-motor neuron. The raw data underlying these figures can be found in S10 Data. (PDF) [file pbio.3003030.s008.pdf]
