# Supplementary material for: Members of the DIP and Dpr adhesion protein families use cis inhibition to shape neural development in Drosophila
Source: PLoS Biol. 2025 Mar 3;23(3):e3003030. doi: 10.1371/journal.pbio.3003030 (PMC12135937; doi:10.1371/journal.pbio.3003030)
Supplement: S5 Fig — (A) Schematic demonstrating single amino acid deletion method of searching for GPI anchor. (B) Representative output for GPI anchor search. (C, D) Net GPI outputs for different DIP-β inputs. (PDF) [file pbio.3003030.s005.pdf]

A

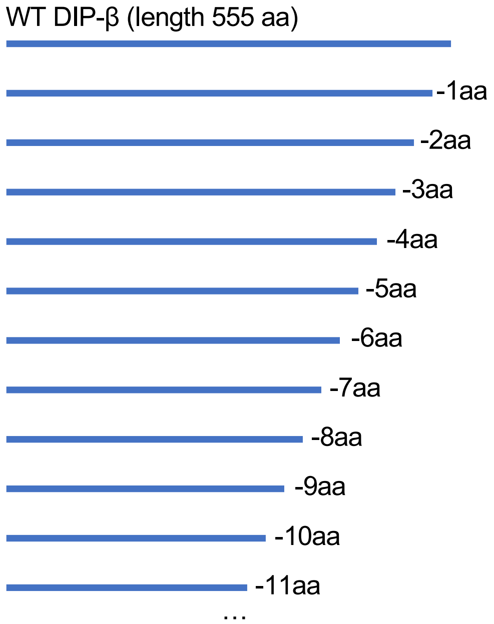

B

# NetGPI 1.1					
**# ID**	**Seq-length**	**Pred. GPI-Anchored**	**Omega-site pos.**	**Likelihood**	**Amino-acid**
DIPbeta_Drosophila_melanogaster_gi221500550_555	555	Not GPI-Anchored	-	0.941	*
DIPbeta_Drosophila_melanogaster_gi221500550_554	554	Not GPI-Anchored	-	0.951	*
DIPbeta_Drosophila_melanogaster_gi221500550_553	553	Not GPI-Anchored	-	0.931	*
DIPbeta_Drosophila_melanogaster_gi221500550_552	552	Not GPI-Anchored	-	0.942	*
DIPbeta_Drosophila_melanogaster_gi221500550_551	551	Not GPI-Anchored	-	0.965	*
DIPbeta_Drosophila_melanogaster_gi221500550_550	550	Not GPI-Anchored	-	0.927	*
DIPbeta_Drosophila_melanogaster_gi221500550_549	549	Not GPI-Anchored	-	0.930	*
DIPbeta_Drosophila_melanogaster_gi221500550_548	548	Not GPI-Anchored	-	0.954	*
DIPbeta_Drosophila_melanogaster_gi221500550_547	547	Not GPI-Anchored	-	0.902	*
…	546-502	Not GPI-Anchored	-	…	*
DIPbeta_Drosophila_melanogaster_gi221500550_501	501	Not GPI-Anchored	-	0.531	*
DIPbeta_Drosophila_melanogaster_gi221500550_500	500	Not GPI-Anchored	-	0.387	*
DIPbeta_Drosophila_melanogaster_gi221500550_499	499	GPI-Anchored	458	0.444	S
DIPbeta_Drosophila_melanogaster_gi221500550_498	498	GPI-Anchored	458	0.476	S
DIPbeta_Drosophila_melanogaster_gi221500550_497	497	GPI-Anchored	458	0.487	S
DIPbeta_Drosophila_melanogaster_gi221500550_496	496	GPI-Anchored	458	0.476	S
DIPbeta_Drosophila_melanogaster_gi221500550_495	495	GPI-Anchored	458	0.487	S
DIPbeta_Drosophila_melanogaster_gi221500550_494	494	GPI-Anchored	458	0.503	S
DIPbeta_Drosophila_melanogaster_gi221500550_493	493	GPI-Anchored	458	0.505	S
DIPbeta_Drosophila_melanogaster_gi221500550_492	492	GPI-Anchored	458	0.510	S
DIPbeta_Drosophila_melanogaster_gi221500550_491	491	GPI-Anchored	458	0.512	S
DIPbeta_Drosophila_melanogaster_gi221500550_490	490	GPI-Anchored	458	0.507	S
DIPbeta_Drosophila_melanogaster_gi221500550_489	489	GPI-Anchored	458	0.511	S
DIPbeta_Drosophila_melanogaster_gi221500550_488	488	GPI-Anchored	458	0.516	S
DIPbeta_Drosophila_melanogaster_gi221500550_487	487	GPI-Anchored	458	0.511	S
DIPbeta_Drosophila_melanogaster_gi221500550_486	486	GPI-Anchored	458	0.517	S
DIPbeta_Drosophila_melanogaster_gi221500550_485	485	GPI-Anchored	458	0.524	S
DIPbeta_Drosophila_melanogaster_gi221500550_484	484	GPI-Anchored	458	0.531	S
DIPbeta_Drosophila_melanogaster_gi221500550_483	483	GPI-Anchored	458	0.536	S
DIPbeta_Drosophila_melanogaster_gi221500550_482	482	GPI-Anchored	458	0.543	S
DIPbeta_Drosophila_melanogaster_gi221500550_481	481	GPI-Anchored	458	0.555	S
DIPbeta_Drosophila_melanogaster_gi221500550_480	480	GPI-Anchored	458	0.569	S
DIPbeta_Drosophila_melanogaster_gi221500550_479	479	GPI-Anchored	458	0.586	S
DIPbeta_Drosophila_melanogaster_gi221500550_478	478	GPI-Anchored	458	0.592	S
DIPbeta_Drosophila_melanogaster_gi221500550_477	477	GPI-Anchored	458	0.601	S
DIPbeta_Drosophila_melanogaster_gi221500550_476	476	Not GPI-Anchored	-	0.461	*

C

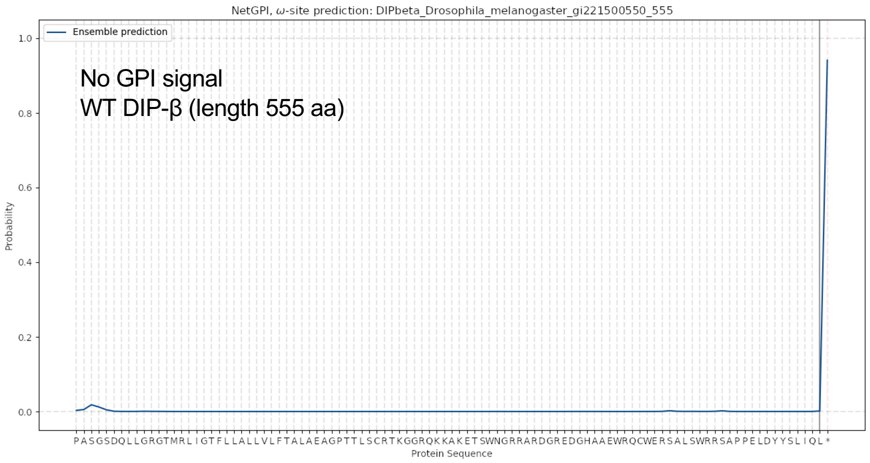

D

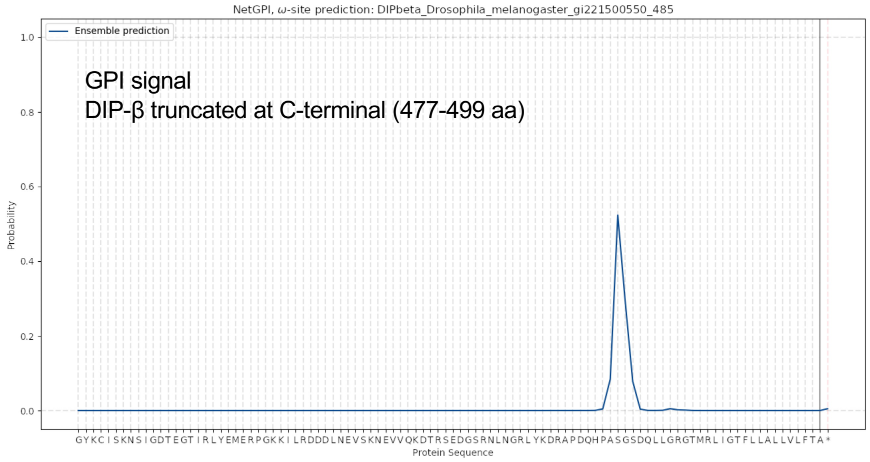

Figure S5.
