# Supplementary material for: Members of the DIP and Dpr adhesion protein families use cis inhibition to shape neural development in Drosophila
Source: PLoS Biol. 2025 Mar 3;23(3):e3003030. doi: 10.1371/journal.pbio.3003030 (PMC12135937; doi:10.1371/journal.pbio.3003030)
Supplement: S6 Fig — (A) Anticipated amino acid characteristics of the GPI-anchored protein. (B) Multiple sequence alignment (MSA) of canonical isoforms of selected DIP and Dpr C-terminal regions across various Drosophila species; ω-sites highlighted in red and the potential hydrophobic region in orange. Alignment executed via Clustal Omega and visualization achieved with Jalview. The coloring in the MSA reflects sequence identity, with deeper shades of blue indicating higher levels of sequence conservation. (PDF) [file pbio.3003030.s006.pdf]

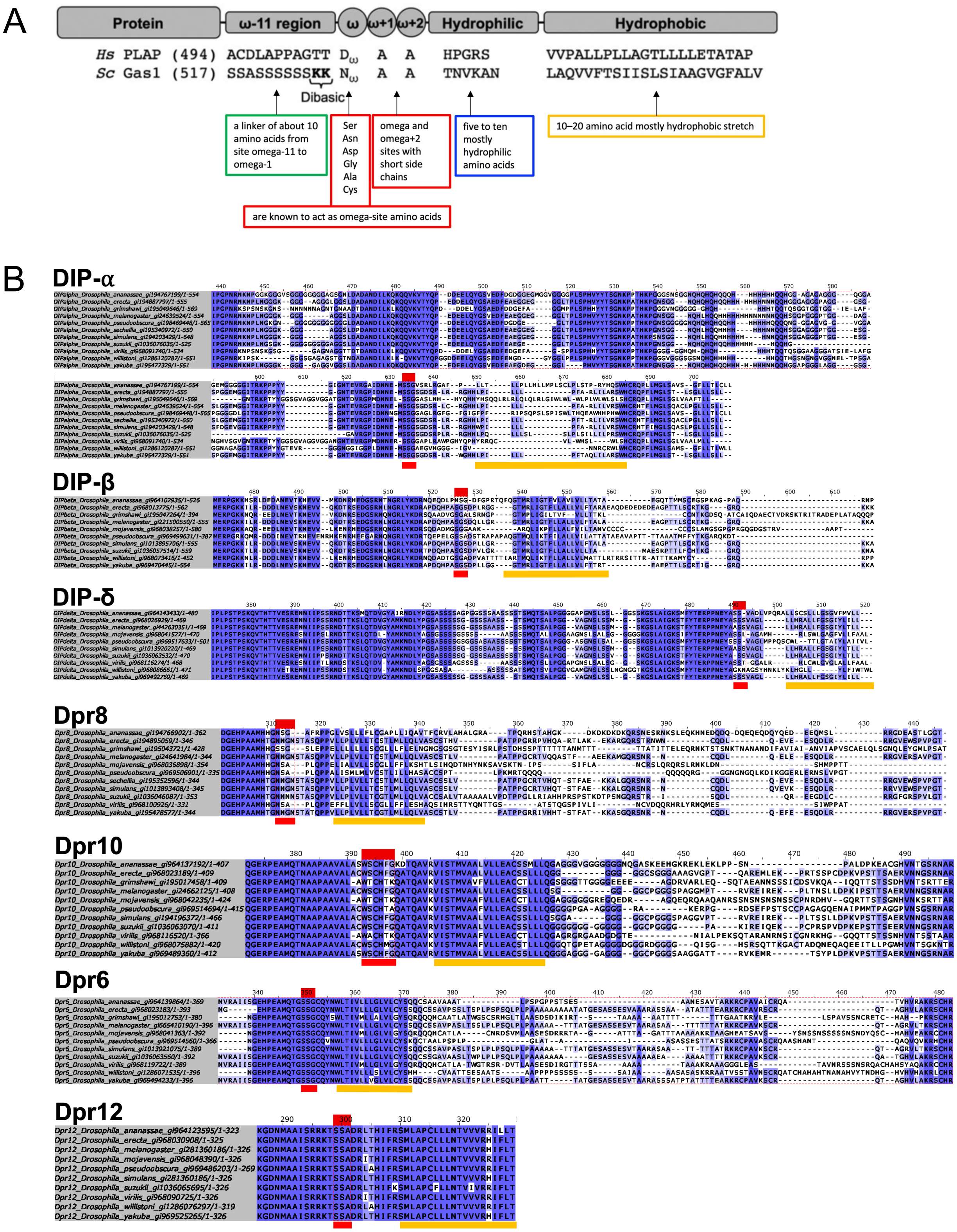
A
Protein
ω-11 region
ω
ω+1
ω+2
Hydrophilic
Hydrophobic
Hs PLAP (494) ACDLAPPAGTT Dω A A HPGRS VVPALLPLLAGTLLLLETATAP
Sc Gas1 (517) SSASSSSSSKK Nω A A TNVKAN LAQVVFTSIISLSIAAGVGFALV
Dibasic
a linker of about 10 amino acids from site omega-11 to omega-1
Ser Asn Asp Gly Ala Cys
omega and omega+2 sites with short side chains
five to ten mostly hydrophilic amino acids
10–20 amino acid mostly hydrophobic stretch
are known to act as omega-site amino acids
B
DIP-α
DIP-β
DIP-δ
Dpr8
Dpr10
Dpr6
Dpr12

Figure S6:
