# Supplementary material for: Members of the DIP and Dpr adhesion protein families use cis inhibition to shape neural development in Drosophila
Source: PLoS Biol. 2025 Mar 3;23(3):e3003030. doi: 10.1371/journal.pbio.3003030 (PMC12135937; doi:10.1371/journal.pbio.3003030)
Supplement: S1 Table — (DOCX) [file pbio.3003030.s009.docx]

Supplemental Data Table I

|  | DIP-α | DIP-β-C | DIP-β-G | DIP-δ | DIP-ε | DIP-ζ |
| --- | --- | --- | --- | --- | --- | --- |
| Dpr6 | 2.06 | 19.4 | 15.25 | >300 | 210 | 151 |
| Dpr8 | >500 | 1.52 | .789 | >500 | >1000 | >500 |
| Dpr10-A | 1.34 | 84 | 5.76 | unknown | unknown | unknown |
| Dpr10-D | 1.67 | 54.9 | 33.3 | 218 | >1000 | >500 |
| Dpr12 | >1000 | >500 | unknown | 2.44 | >1000 | >500 |

Table I- K_D_s important for this study

K_D_s are indicated in micromolar and appropriate references are shown below

Reported in this study (S2 and S3 Figs)

Reported in Cosmanescu, et. al, 2018 ^9^

Reported in Carrillo, et. al, 2015 ^3^

Reported in Sergeeva, et. al, 2020 ^11^
