# Supplementary material for: Members of the DIP and Dpr adhesion protein families use cis inhibition to shape neural development in Drosophila
Source: PLoS Biol. 2025 Mar 3;23(3):e3003030. doi: 10.1371/journal.pbio.3003030 (PMC12135937; doi:10.1371/journal.pbio.3003030)
Supplement: S3 Table — (DOCX) [file pbio.3003030.s011.docx]

| **Reagent type  (species) or  resource** | **Designation** | **Source or reference** | **Additional information** |
| --- | --- | --- | --- |
| Genetic reagent  (D. melanogaster) | *DIP-α-T2A-Gal4* | Venkatasubramanian et. al 2019 ^12^ |  |
| Genetic reagent  (D. melanogaster) | *UAS-dpr6-V5* | Xu et. al. 2018 ^13^ | Provided by K. Zinn |
| Genetic reagent  (D. melanogaster) | *Mef2-Gal4* |  | Bellen Lab, provided  by R.Carrillo |
| Genetic reagent  (D. melanogaster) | *DIP-α-T2A-QF* | Venkatasubramanian et. al 2019 ^12^ |  |
| Genetic reagent  (D. melanogaster) | *UAS-dpr10-V5* | Xu et. al. 2018 ^13^ | Provided by K. Zinn |
| Genetic reagent  (D. melanogaster) | *A8-Gal4* | Venkatasubramanian et. al 2019 ^12^ |  |
| Genetic reagent  (D. melanogaster) | *UAS-dpr10-RNAi* | BDSC #27991 |  |
| Genetic reagent  (D. melanogaster) | *UAS-dpr6-RNAi* | VDRC#103521 |  |
| Genetic reagent  (D. melanogaster) | *20XUAS-6X-GFP* | BDSC #52261;  BDSC #52262 |  |
| Genetic reagent  (D. melanogaster) | *10XQUAS-6XGFP* | BDSC #52264 |  |
| Genetic reagent  (D. melanogaster) | *UAS-DIP-α* | Xu et. al. 2018 | Generated by the Zipursky Lab |
| Genetic reagent  (D. melanogaster) | *DIP-α^short^* | This paper |  |
| Genetic reagent  (D. melanogaster) | *UAS-DIP-δ* | Bornstein et al., 2021 ^14^ |  |
| Genetic reagent  (D. melanogaster) | *U*AS*-Dpr12* | Bornstein et al., 2021 ^14^ |  |
| Genetic reagent  (D. melanogaster) | *dpr12^Δ50-81^* | Bornstein et al., 2021 ^14^ |  |
| Genetic reagent  (D. melanogaster) | *GMR71G10-Gal4* | BDSC #39604 |  |
| Genetic reagent  (D. melanogaster | *10XUAS-IVS-nCD8::GFP* (on III) | BDSC #32187 |  |
| Genetic reagent  (D. melanogaster | *10XUAS-IVS-nCD8::GFP* (on II) | BDSC #32186 |  |

Supplementary Data File 3: Fly strains used in this paper.
