# Supplementary material for: Members of the DIP and Dpr adhesion protein families use cis inhibition to shape neural development in Drosophila
Source: PLoS Biol. 2025 Mar 3;23(3):e3003030. doi: 10.1371/journal.pbio.3003030 (PMC12135937; doi:10.1371/journal.pbio.3003030)
Supplement: Supplementary file 6 [file pbio.3003030.s010.docx]

Accession	Localization	Position	Score
Dpr10_Drosophila_melanogaster_gi24662125_408	No GPI-anchor found	-	0
Dpr10_Drosophila_melanogaster_gi24662125_407	No GPI-anchor found	-	0
Dpr10_Drosophila_melanogaster_gi24662125_406	No GPI-anchor found	-	0
Dpr10_Drosophila_melanogaster_gi24662125_405	No GPI-anchor found	-	0
Dpr10_Drosophila_melanogaster_gi24662125_404	No GPI-anchor found	-	0
Dpr10_Drosophila_melanogaster_gi24662125_403	No GPI-anchor found	-	0
Dpr10_Drosophila_melanogaster_gi24662125_402	No GPI-anchor found	-	0
Dpr10_Drosophila_melanogaster_gi24662125_401	No GPI-anchor found	-	0
Dpr10_Drosophila_melanogaster_gi24662125_400	No GPI-anchor found	-	0
Dpr10_Drosophila_melanogaster_gi24662125_399	No GPI-anchor found	-	0
Dpr10_Drosophila_melanogaster_gi24662125_398	No GPI-anchor found	-	0
Dpr10_Drosophila_melanogaster_gi24662125_397	No GPI-anchor found	-	0
Dpr10_Drosophila_melanogaster_gi24662125_396	No GPI-anchor found	-	0
Dpr10_Drosophila_melanogaster_gi24662125_395	No GPI-anchor found	-	0
Dpr10_Drosophila_melanogaster_gi24662125_394	No GPI-anchor found	-	0
Dpr10_Drosophila_melanogaster_gi24662125_393	No GPI-anchor found	-	0
Dpr10_Drosophila_melanogaster_gi24662125_392	No GPI-anchor found	-	0
Dpr10_Drosophila_melanogaster_gi24662125_391	No GPI-anchor found	-	0
Dpr10_Drosophila_melanogaster_gi24662125_390	No GPI-anchor found	-	0
Dpr10_Drosophila_melanogaster_gi24662125_389	No GPI-anchor found	-	0
Dpr10_Drosophila_melanogaster_gi24662125_388	No GPI-anchor found	-	0
Dpr10_Drosophila_melanogaster_gi24662125_387	No GPI-anchor found	-	0
Dpr10_Drosophila_melanogaster_gi24662125_386	No GPI-anchor found	-	0
Dpr10_Drosophila_melanogaster_gi24662125_385	No GPI-anchor found	-	0
Dpr10_Drosophila_melanogaster_gi24662125_384	No GPI-anchor found	-	0
Dpr10_Drosophila_melanogaster_gi24662125_383	No GPI-anchor found	-	0
Dpr10_Drosophila_melanogaster_gi24662125_382	No GPI-anchor found	-	0
Dpr10_Drosophila_melanogaster_gi24662125_381	No GPI-anchor found	-	0
Dpr10_Drosophila_melanogaster_gi24662125_380	No GPI-anchor found	-	0
Dpr10_Drosophila_melanogaster_gi24662125_379	No GPI-anchor found	-	0
Dpr10_Drosophila_melanogaster_gi24662125_378	No GPI-anchor found	-	0
Dpr10_Drosophila_melanogaster_gi24662125_377	No GPI-anchor found	-	0
Dpr10_Drosophila_melanogaster_gi24662125_376	No GPI-anchor found	-	0
Dpr10_Drosophila_melanogaster_gi24662125_375	No GPI-anchor found	-	0
Dpr10_Drosophila_melanogaster_gi24662125_374	No GPI-anchor found	-	0
Dpr10_Drosophila_melanogaster_gi24662125_373	No GPI-anchor found	-	0
Dpr10_Drosophila_melanogaster_gi24662125_372	No GPI-anchor found	-	0
Dpr10_Drosophila_melanogaster_gi24662125_371	No GPI-anchor found	-	0
Dpr10_Drosophila_melanogaster_gi24662125_370	No GPI-anchor found	-	0
Dpr10_Drosophila_melanogaster_gi24662125_369	No GPI-anchor found	-	0
Dpr10_Drosophila_melanogaster_gi24662125_368	No GPI-anchor found	-	0
Dpr10_Drosophila_melanogaster_gi24662125_367	No GPI-anchor found	-	0
Dpr10_Drosophila_melanogaster_gi24662125_366	No GPI-anchor found	-	0
Dpr10_Drosophila_melanogaster_gi24662125_365	No GPI-anchor found	-	0
Dpr10_Drosophila_melanogaster_gi24662125_364	No GPI-anchor found	-	0
Dpr10_Drosophila_melanogaster_gi24662125_363	No GPI-anchor found	-	0
Dpr10_Drosophila_melanogaster_gi24662125_362	No GPI-anchor found	-	0
Dpr10_Drosophila_melanogaster_gi24662125_361	No GPI-anchor found	-	0
Dpr10_Drosophila_melanogaster_gi24662125_360	No GPI-anchor found	-	0
Dpr10_Drosophila_melanogaster_gi24662125_359	No GPI-anchor found	-	0
Dpr10_Drosophila_melanogaster_gi24662125_358	No GPI-anchor found	-	0
Dpr10_Drosophila_melanogaster_gi24662125_357	GPI-anchor	326	0.7
Dpr10_Drosophila_melanogaster_gi24662125_356	GPI-anchor	325	1
Dpr10_Drosophila_melanogaster_gi24662125_355	GPI-anchor	324	1
Dpr10_Drosophila_melanogaster_gi24662125_354	GPI-anchor	323	1
Dpr10_Drosophila_melanogaster_gi24662125_353	GPI-anchor	322	1
Dpr10_Drosophila_melanogaster_gi24662125_352	GPI-anchor	321	1
Dpr10_Drosophila_melanogaster_gi24662125_351	GPI-anchor	320	1
Dpr10_Drosophila_melanogaster_gi24662125_350	GPI-anchor	321	1
Dpr10_Drosophila_melanogaster_gi24662125_349	GPI-anchor	321	1
Dpr10_Drosophila_melanogaster_gi24662125_348	GPI-anchor	322	1
Dpr10_Drosophila_melanogaster_gi24662125_347	GPI-anchor	321	1
Dpr10_Drosophila_melanogaster_gi24662125_346	GPI-anchor	321	1
Dpr10_Drosophila_melanogaster_gi24662125_345	GPI-anchor	320	1
Dpr10_Drosophila_melanogaster_gi24662125_344	GPI-anchor	321	1
Dpr10_Drosophila_melanogaster_gi24662125_343	GPI-anchor	320	1
Dpr10_Drosophila_melanogaster_gi24662125_342	GPI-anchor	321	0.7
Dpr10_Drosophila_melanogaster_gi24662125_341	GPI-anchor	321	0.55
Dpr10_Drosophila_melanogaster_gi24662125_340	No GPI-anchor found	-	0
Dpr10_Drosophila_melanogaster_gi24662125_339	No GPI-anchor found	-	0
Dpr10_Drosophila_melanogaster_gi24662125_338	No GPI-anchor found	-	0
Dpr10_Drosophila_melanogaster_gi24662125_337	No GPI-anchor found	-	0
Dpr10_Drosophila_melanogaster_gi24662125_336	No GPI-anchor found	-	0
Dpr10_Drosophila_melanogaster_gi24662125_335	No GPI-anchor found	-	0
